# Research progress on the application of organoids in gynecological tumors

**DOI:** 10.3389/fphar.2024.1417576

**Published:** 2024-06-26

**Authors:** Ying Shen, Yu Wang, Si-yu Wang, Chan Li, Feng-Juan Han

**Affiliations:** ^1^ The First School of Clinical Medicine, Heilongjiang University of Chinese Medicine, Harbin, China; ^2^ Department of Obstetrics and Gynecology, The First Affiliated Hospital of Heilongjiang University of Chinese Medicine, Harbin, China

**Keywords:** gynecological tumors, organoid, ovarian cancer, endometrial cancer, cervical cancer, tumor models

## Abstract

Organoids are *in vitro* 3D models that maintain their own tissue structure and function. They largely overcome the limitations of traditional tumor models and have become a powerful research tool in the field of oncology in recent years. Gynecological malignancies are major diseases that seriously threaten the life and health of women and urgently require the establishment of models with a high degree of similarity to human tumors for clinical studies to formulate individualized treatments. Currently, organoids are widely studied in exploring the mechanisms of gynecological tumor development as a means of drug screening and individualized medicine. Ovarian, endometrial, and cervical cancers as common gynecological malignancies have high morbidity and mortality rates among other gynecological tumors. Therefore, this study reviews the application of modelling, drug efficacy assessment, and drug response prediction for ovarian, endometrial, and cervical cancers, thereby clarifying the mechanisms of tumorigenesis and development, and providing precise treatment options for gynecological oncology patients.

## 1 Introduction

Cervical cancer (CC), endometrial cancer (EC), and ovarian cancer (OC) are known as the three major malignant tumors in gynecology, which are silent killers of women’s lives and health worldwide. Inhibiting the occurrence and development of the three major malignant tumors in gynecology is a major challenge currently facing the medical field. Organoid is an emerging *in vitro* 3D modeling technology with the characteristics of reproducing the physiological and pathological features of the original tissues *in vivo*, which has shown good applicability in the fields of disease modeling, regeneration mechanism, and precision medicine. Therefore, further thinking about the integration of gynecological malignancies and organoids, with modeling approach as the starting point and drug efficacy assessment and drug response prediction as the endpoints for research, may help to provide a new strategy of targeted drug delivery for the treatment of gynecological malignancies featuring precision and personalization. Gynecological malignant tumors are major diseases that pose a serious threat to women’s lives and health worldwide. Studies have shown that the incidence and mortality rates of common gynecological malignant tumors such as CC, EC, and OC have increased rapidly in recent years (Miller et al., 2019). Gynecological malignancies place a heavy medical burden on women’s overall health, with uterine, cervical, and ovarian cancers causing approximately 30,000 deaths per year in the United States (Ferlay et al., 2015). In the conventional treatment of gynecological malignancies, surgery plus chemotherapy is the treatment of choice for most patients. However, standard therapies are not applicable to every patient; therefore, there is an urgent need for effective methods to understand the tumor characteristics of patients to perform predictive analyses of tumors and provide more personalized and precise treatment options (Wahida et al., 2023).

Cell cultures and animal models are the experimental cornerstones of the medical field for the study of tissues, organs, and body physiology. However, their ability to accurately reflect human mechanisms *in vivo* is limited. Organoids are micro-organs with three-dimensional (3D) structures that can highly mimic the morphology and physiological functions of the source tissues and produce tissues with stable phenotypes, making them good models for clinical disease research (Semertzidou et al., 2020). As research continues to evolve, human-derived models have progressed from monolayer cell cultures to 3D organoids, and cultivating 3D organoids can be used to explore the mechanisms of disease occurrence and development. Tumor organoids have become the dominant experimental model for exploring the diagnosis and treatment of tumor diseases, and can be used to analyze the genetic or epigenetic basis, uncover drug resistance mechanisms, conduct drug screening, and individualize treatment for patients, so this direction is a breakthrough point, and it is hoped that it will enhance the prospects for personalized medicine based on the study of organoids of patient origin (Driehuis et al., 2020; Li et al., 2021; Shamshirgaran et al., 2021; Wu et al., 2021; Wang et al., 2023).

Previous studies have shown that organoids can reproduce the biological characteristics and tumor heterogeneity of primary tissues, making them a novel and reliable clinical model in the study of gynecological malignant tumors (Sachs et al., 2018; Kopper et al., 2019; Yoon et al., 2021). Kopper et al. established 56 organoid lineages from OC tissue and tested their sensitivity to commonly used platinum or paclitaxel drugs in OC treatment regimens. Their results showed that the HGS-3.1 organoid lineage was highly sensitive to drugs such as gemcitabine, carboplatin, and paclitaxel, while exhibiting resistance to drugs targeting the PI3K/AKT/mTOR pathway (Kopper et al., 2019). McDowell et al. evaluated the activity of artemisinin in primary OC organoids and found that artesunate treatment can induce G1 phase arrest, upregulate G1/S conversion, and reduce cell viability in OC cell models (McDowell et al., 2021). Artesunate combined with carboplatin and paclitaxel can enhance its effectiveness and enhance clinical efficacy (McDowell et al., 2021). Bi et al. established organoid models of EC and OC tissues from patient sources, tested the most used drugs, and found that these models reflected a series of sensitivities to platinum-containing chemotherapy and other related drugs (Bi et al., 2021). They successfully predicted the organoid postoperative chemotherapy and trastuzumab resistance of patients receiving neoadjuvant trastuzumab treatment before surgery, suggesting that organoids can be used as a preclinical platform for personalized cancer treatment in patients with gynecological cancer (Bi et al., 2021). Therefore, organoids not only play an important role in exploring signaling pathways, gene screening, and other mechanisms of tumor occurrence and development in the field of gynecological cancer but also provide a platform for mutation carcinogenesis modeling, drug screening, patient stratification, and drug response prediction. This fills the gap between basic research and clinical practice and helps with personalized treatment (Semertzidou et al., 2020).

In summary, it can be found that organoids have been widely used in the research of gynecological tumors. With this background, we reviewed common gynecological tumors such as OC, EC, and CC and analyzed the application research progress of organoids in gynecological tumors from the perspectives of modeling, drug screening, and drug evaluation to provide directions for future research.

## 2 Ovarian cancer organoids

Among all gynecological malignancies, OC has the highest mortality rate owing to high treatment resistance, long incubation period, and lack of effective treatment methods (Torre et al., 2018; Siegel et al., 2020). This requires preclinical models to summarize the histological, molecular, and pathophysiological characteristics of different subtypes of OC. Furthermore, OC is a heterogeneous disease composed of a series of subtypes, among which epithelial type is the most common. Given the significant heterogeneity of OC, the same treatment method can produce different clinical outcomes in individual patients (Kurman and Shih Ie, 2016). For example, poly ADP ribose (PARP) inhibitors are effective in patients with homologous recombination deficiency (HRD), but ineffective in patients without HR deficiency (Yang et al., 2021). Therefore, there is an urgent need for promising preclinical models to achieve precision medicine. Organoids, as models from patients, can address the heterogeneity of OC and simulate the progression of OC in patients, providing important insights for personalized treatment.

### 2.1 Modeling of OC organoids

At present, research has successfully developed various histological subtypes of organoids, including serous OC organoids, epithelial OC organoids, clear cell carcinoma, and endometrioid OC organoids (Nanki et al., 2020; Wan et al., 2023; Wu et al., 2023). By constructing an OC disease model, capturing the characteristics of histological cancer subtypes, replicating the mutation landscape of the primary tumor, and preserving the genomic map of the originating tumor, the usefulness of identifying genes related to OC progression can contribute to the study of disease etiology (Iwahashi et al., 2022). Among them, serous OC organoids are the current research focus. The following shows the modeling methods for ovarian cancer organoids with different tissue types.

#### 2.1.1 Serous OC organoids

The matrix gel method is currently the main method for culturing serous OC organoids, with the main difference being the source of the extracted samples. Raab et al. extracted samples from patients with epithelial OC and cultured serous OC organoids using the matrix gel method. They found that restoring p53 function can inhibit the proliferation of advanced serous ovarian cancer (HGSOC) cells, reduce chromosomal instability, and cause cell death (Raab et al., 2024). Iwahashi et al. extracted samples from 121 patients with wild-type p53 or mutant p53 high-grade serous OC and constructed OC organoids using the matrix gel method. They found that mutant p53 aggregates hindered the apoptotic function of wild-type p53 in recipient cells, and p53 aggregation inhibitors restored cell apoptosis in tumor organoids derived from patients carrying p53 aggregates, which is an appropriate p53 function (Iwahashi et al., 2022). The transmission of p53 aggregates is related to poor cancer prognosis and chemotherapy resistance (Iwahashi et al., 2022). Carvalho et al. drained ascites or pleural effusion from patients diagnosed with serous OC and cultured them into organoids using the matrix gel method. The study found that ligands and receptors of the PI3K-AKT pathway are important mediators for cancer-associated fibroblasts (CAFs) to communicate with other cancer cells (Carvalho et al., 2022). Compadre et al. constructed organoids from tumor biopsies or malignant ascites collected from patients with HGSOC by using the matrix gel method and showed that DNA repair protein RAD51 scores of platinum non-responding tumor organoids were significantly higher than those of platinum-responsive tumor organoids, and that the RAD51 foci were a potent marker of response to platinum-based chemotherapy and survival in OC (Compadre et al., 2023). Cao et al. cultured a high-grade plasmacytoid carcinoma organoid model that was highly similar to clinical OC tissues (Cao et al., 2023). Clinical OC tissues extracted from patients were used to culture the organoids by using the matrix gel method. After development of the organoids, they were passaged every 15 days, and the culture was successful when the fragments were rounded and showed features of the parental tumor within 7 days.

On the basis of the matrix gel method, some studies have attempted to improve the cultivation method or tissue source. Wang et al. quickly transferred fresh high-grade serous cancer tissue into the cell culture room after surgery and used matrix gel method to form organoids. The improvement in this study was the addition of 500 μL advanced DMEM/F12 medium containing specific growth factors along with 10 mmol/L niacinamide, and it was found that the expression of FBN1 was significantly enhanced in cisplatin-resistant OC-like organs and tissues, which may be a key factor in chemotherapy resistance (Wang Z. et al., 2022). Lõhmussaar et al. established an *in vitro* tumor model of fallopian tubes and ovarian surface epithelium (OSE) by targeting the mouse *Trp53* gene alone or in combination with *Brca1*, *Pten*, and *Nf1*. The mouse fallopian tubes and OSE tissues were dissected, and organoids were constructed using the matrix gel method. Additionally, the addition of FGF2 during the initial passage period in the culture medium of the OSE organoids improved the growth of organoids (Lõhmussaar et al., 2020b). By cultivating these two types of organs, it has been proven that both the surface epithelial cells of the fallopian tubes and ovaries can cause high-grade serous OC. Maenhoudt et al. established organoids by using tissue derived from HGSOC patients by the matrix gel method. Furthermore, by testing a variety of media components, it was ultimately determined that the addition of neuromodulin-1 (NRG1) was the optimal key factor to enable the development and growth of OC organoids (Maenhoudt et al., 2020). Cesari et al. collected biopsies from patients, placed them in culture dishes containing AdDF+++medium, chopped them, and digested them in AdDF+++medium containing RHO/ROCK pathway inhibitors to form high-grade serous cancerous organoids from the patient’s source through matrix gel method (Cesari et al., 2023). Wang et al. extracted tissues from 20 patients with serous OC, cut them into small pieces, and incubated them in dispersed enzymes. Then, undigested tissue fragments were filtered and removed and embedded in a matrix gel growth factor-reduced basement membrane matrix without phenol red, ultimately forming OC-like organs (Wang W. et al., 2022). Hoffmann et al. obtained specimens from highly purified tumor deposits in the peritoneum or omentum of patients with HGSOC, inoculated in Matrigel matrix gel and supplemented with growth factor cultures to establish omental histologically derived organoids. They found that activation of the Wnt pathway could lead to growth arrest of these organoids and that active BMP signaling was required for the generation of HGSOC organoids (Hoffmann et al., 2020).

In addition to matrix gel method, micro seeding and hydrogel methods are also some of the main methods for serous OC-like organoids. For example, Phan et al. successfully cultured ovarian tumor-derived organoids by using the micro ring seeding method to determine drug sensitivity (Phan et al., 2019). Unlike traditional cultivation methods, by adjusting the geometric shape of tumor cells in the Matrix, single-cell suspensions obtained from cell lines or pre mixed samples with cold matrix gel were plated into a circular shape around the edge of the well plate to generate micro rings around the edge of the well. The results showed that compared with traditional cultivation methods, the micro ring seeding method not only had no impact on growth and drug treatment but could also perform automated high-throughput screening (Phan et al., 2019). Pietilä et al. used the hydrogel method to construct OC-like organs, prepared a hydrogel rich in laminin, and screened the 3D culture cell model from the extracellular matrix (Pietilä et al., 2021). To evaluate the chemical resistance of high-grade serous OC in a tissue-like environment, freshly isolated HGSC cell clusters derived from ascites from patients who underwent one tumor removal surgery or neoadjuvant chemotherapy were treated in COL1 or COL1+COL6 medium for 4 days, followed by 72 h of treatment. After 4 days of 3D culture, these short-term organoids exhibited HGSC morphology, invasive growth in collagen 1 (COL1), and were positive for PAX8 and CK7. COL6 reduced the content of relatively metabolically active cells in 2/4r HGSC-like organs, while in p-HGSC, the activity remained unchanged between matrices or increased + COL in COL1. Notably, among all r-HGSC short-term organoids, COL6 confers cisplatin resistance, while p-HGSC is not affected by treatment and may even become increasingly sensitive. In summary, these results indicate that cisplatin enhances the adhesion of COL6, and COL6 enhances specific protection against cisplatin cytotoxicity in r-HGSC cells. In addition, the protective effect of COL6 can be derived from the intrinsic platinum-resistance mechanism, which is already active in HGSC cells derived from recurrent diseases. It has also been proven that gradually altering the intrinsic adhesion signal transduction and surrounding the extracellular matrix (ECM) of cancer cells to enhance platinum chemotherapy itself can enhance drug resistance. Specific ECM can be achieved through Focal Adhesion Kinase (FAK), and β1 whole protein pMLC-YAP signaling pathway increases resistance to platinum-mediated apoptosis–induced DNA damage (Pietilä et al., 2021).

#### 2.1.2 Other types of OC organoids

For other types of OC models, the matrix gel method is still a commonly used culture method. Zhang et al. obtained ovarian tumor tissues from patients undergoing surgery and established ovarian epithelial cancerous organoids using the matrix gel method. This study observed the morphology of initial organoid cell clusters on a daily basis (Zhang L. et al., 2023). Given the fact that OC organoids originate from different patients, there is significant tumor heterogeneity. The initial OC organoid cell clusters will gradually form OC organoids, which can exhibit cystic or solid growth under light microscopy and gradually increase in volume under 3D culture. By verifying the morphological structure of OC organoids and the expression of molecular markers, the 3D organoid cell viability staining method is used to detect the activity status of OC organoid cells (Zhang L. et al., 2023). Wu et al. obtained OC tissue through biopsy of OC patients and established OC organoids using the matrix gel method. To explore the impact of OC on neutrophils, researchers established a co-culture system of neutrophils and OC-like organs, which is an *in vitro* model that can effectively simulate tumors *in vivo*. By constructing OC-like organs to stimulate neutrophils, the final results showed that MUC16 (CA125) induced neutrophil inflammatory response, thereby promoting the development of systemic excessive inflammation in patients with OC. The factors upregulated by neutrophil inflammatory response can lead to immunosuppressive tumor microenvironment and inhibit NK cells (Wu et al., 2023).

In addition, the simultaneous preparation of various types of OC organoids for comparative research is also a current trend. For example, Kawata et al. prepared OC organoids using matrix gel method, which were prepared from surgical excess or abdominal fluid from OC patients from clinical specimens, including excess abdominal fluid from OC patients during surgery. This study investigated the role of the top basal polarity of OC cell clusters in peritoneal dissemination by utilizing OC organoids from various histological types (Kawata et al., 2022). It was found that polarity switching mediated by SRC family kinases (SFK) is associated with peritoneal metastasis, and polarity switching will be a potential therapeutic target for inhibiting peritoneal dissemination of OC (Kawata et al., 2022). Nanki et al. established different histological subtypes (HGSC, EM, CCC) of organs using the matrix gel method. It was found that organoids cultured with cocktail medium are most effective for multi tissue culture. The overall success rate of organoid culture was 80%, ultimately capturing the histological features and p53 positivity of the primary tumor (Nanki et al., 2020).

### 2.2 Drug testing and screening

In the past few years, the application of OC organoids in promoting precision medicine, drug detection, and screening is rapidly expanding. Previous studies have mainly focused on gene modification of OC organoids, or constructing organoids using tissues with different gene mutations to construct different OC disease models for drug efficacy evaluation, drug screening experiments, or drug sensitivity testing (Wang W. et al., 2022; Liu et al., 2022; Cao et al., 2023).

#### 2.2.1 Drug efficacy evaluation

Cesari et al. evaluated the drug efficacy of THZ531, a CDK12/13 inhibitor, using organoids and analyzed its effects on HGSOC cells and patient-derived organoids. The results confirmed the strong anti-cancer activity of cyclin-dependent kinase 12 and 13 (CDK12/13) inhibitors and the synergistic effect of THZ531 and pathway inhibitors (EGFR, RPTOR, ATRIP) regulated by cancer-related genes on the activity of HGSOC organoids (Cesari et al., 2023). Wang et al. utilized organoids to investigate Wnt/β-catenin. The efficacy evaluation of the serial protein inhibitor CWP232291 was conducted, and CWP232291 inhibited by β-chain proteins significantly slowed down the growth of OC and could also inhibit the growth of cisplatin-resistant cell lines and organoids derived from patients with serous OC patients (Wang W. et al., 2022).

#### 2.2.2 Drug screening test

The organoid derived from the patient is a suitable *in vitro* model that can be used to screen OC drugs. Zhang et al. used OC-like organs to study the mechanism of acquired resistance to olapanide, a polyADP ribose polymerase inhibitor (PARPi) (Zhang X. et al., 2023). They induced the formation of polyploid giant cancer cells (PGCCs) in ovarian and breast cancer cell lines, organs derived from high-grade serous cancer (HGSC), and patient-derived xenografts (PDX). The results showed that mifepristone blocking the formation of olapanide-induced PGCC could be applied to HGSC-like organ models, and targeting PGC could enhance the therapeutic response to PARPi and overcome PARPi-induced drug resistance (Zhang X. et al., 2023). Cao et al. used OC organoids to study the resistance mechanism and mechanism of action of PARPi, and treated patient-derived organoids with PARPi. PARPi inhibits cell growth by upregulating early cell apoptosis, and PARPi treatment has complex effects on potential gene changes related to PARPi resistance, providing ideas for further research on PARPi resistance mechanisms (Cao et al., 2023). Gray et al. extracted tissue from a patient with platinum-resistant advanced low-grade serous ovarian cancer (LGSOC) who had failed standard chemotherapy and two surgeries. The sample exhibited >70% tumor cell viability, and after 7 days of cultivation using matrix gel method, over 70% pure organoid culture was obtained for drug screening (Gray et al., 2023). After successfully establishing SOC, Vernon et al. identified the broad anti-tumor properties of miR-3622 b-5 p through functional miRNA screening and revealed a new therapeutic combination strategy for ovarian tumor organoids. EGFRi and ABT-737 synergistically act on OC organoids, while the combination of erlotinib and ABT-737 significantly reduces the cell viability of all Patient-derived organoid (Vernon et al., 2020).

#### 2.2.3 Drug sensitivity testing

Ovarian cancer organoids have significant utility in drug sensitivity testing. For example, in the application of SOC organoids, Gorski et al. obtained tissues from OC patients undergoing tumor reduction surgery and developed HGSOC organoids using matrix gel method to test their sensitivity to carboplatin (Gorski et al., 2021). They found that the sensitivity and resistance can be related to the interaction between the NFkB pathway, PRDM6 activation, B-cell receptor signal transduction, and PI3K-AKT signal transduction pathway (Gorski et al., 2021). Lõhmussaar et al. established HGSOC-like organs from mouse fallopian tubes and ovarian epithelial tissues, discovering the different lineage-dependent sensitivities of common drugs in HGSOC (Lõhmussaar et al., 2020b). In the fallopian tube lineage, after obtaining more mutations, cell lines are usually more sensitive to paclitaxel and niraparib. In the OSE lineage, mutant lines showed lower sensitivity to paclitaxel and niraparib. *In vitro* drug testing using organoids showed that patients have different sensitivities to common drugs used to treat HGSOC (Lõhmussaar et al., 2020b). Maenhoudt et al. used HGSOC organoids for screening of nutlin-3 drugs and found that EOC-derived organoids corresponding organoids (EOC-O-7) organoids derived from p53 wild-type tumors are sensitive to nutlin-3, while EOC-O4 and EOC-O8 organoids established by p53 mutant tumors are insensitive to nutlin-3. The study showed that this organoid can simultaneously perform sensitivity testing of multiple nutlin-3 drugs to different types of tissues (Maenhoudt et al., 2020).

In terms of other types of OC organoids, Kopper et al. successfully established epithelial OC organoids for drug sensitivity testing. They tested the sensitivity of OC organoids to platinum or paclitaxel drugs and found that the HGS-3.1 organoid line is highly sensitive to drugs such as gemcitabine, carboplatin, and paclitaxel, while exhibiting resistance to drugs targeting the PI3K/AKT/mTOR pathway (Kopper et al., 2019). Nanki et al. successfully established OC organoids for drug screening and sensitivity testing, including advanced serous, clear cell, and endometrioid cancers. They found that organoids carrying the pathogenic variant of BRCA1 were more sensitive to PARP inhibitor olapanib and platinum-based drugs, while organoids from clear cell OC were resistant to conventional drugs such as OC platinum-based drugs, paclitaxel, and olapanib (Nanki et al., 2020).

### 2.3 Patient stratification and drug response prediction

The inherent molecular heterogeneity is particularly prominent in OC, which leads to differences in drug responses among different patients (Yang et al., 2021). Organ-like models that can preserve the heterogeneity and genetic characteristics of the original tumor have advantages in precision medicine. Organ-like models taken from different patient tissues have different drug responses, and stratifying individual patients into customized plans has broad prospects.

#### 2.3.1 Patient stratification

Sauriol et al. evaluated the response of three high-grade SOC organoids, with one patient resistant to platinum and two patients sensitive to platinum; the latter two received maintenance treatment with olaparib but relapsed, indicating their resistance to olaparib. *In vitro* experiments showed that two models were sensitive to olapanib and one was resistant. The combination of olaparib and nicotinamide phosphoribosyltransferase (NAMPT) inhibitors has a synergistic effect and effectiveness on all three models, including clinically acquired PARPi-resistant models. This study combines PARPi with NAMPT inhibitors and applies them to a PARPi-resistant HGSOC organoid model. The results show that intracellular NAD+is depleted, inducing double stranded DNA breakage and promoting cell apoptosis through caspase-3 cleavage monitoring. In the context of PARPi resistance, NAMPT inhibition provides a promising new option for OC patients (Sauriol et al., 2023).

Gray et al. reported that a platinum-resistant advanced low-grade SOC patient who failed standard chemotherapy and two surgeries rapidly deteriorated into end-of-life care, and genomic analysis of the tumor did not indicate a clear treatment option either. After determining several treatment options through drug sensitivity testing of the patient’s tumor-like organs, the patient achieved remarkable clinical transformation with subsequent treatments, highlighting the clinical efficacy of *in vitro* drug testing of tumor-like organs from the patient’s source as a new functional precision medicine method, which can determine effective personalized therapies for patients who have failed standard care treatments (Gray et al., 2023).

Tao et al. found that among three epithelial OC organoids with homologous recombination repair (HRR) defect mutations, two were sensitive to PARPi and one was congenitally drug-resistant. Another type of organoid derived from relapsed patients during olaparib maintenance therapy was found to develop acquired resistance to PARPi. The subsequent functional analysis revealed potential drug resistance mechanisms associated with replication cross protection and HRR functional recovery. Combination strategies targeting these mechanisms can reverse drug resistance, demonstrating the sensitivity of EOC PDO to PARPi in evaluating different environments (Tao et al., 2022).

#### 2.3.2 Drug reaction prediction

Chen et al. obtained tumor specimens in the form of multicellular spheroids (MCS) from malignant exudates of OC patients and formed organoids through primary culture. It was found that the sensitivity of organoids from different samples of the same patient to carboplatin and paclitaxel chemotherapy drugs varies, and drug sensitivity testing can be conducted through organoids (Chen et al., 2020). After constructing epithelial OC organoids, Zhang et al. added different concentrations of carboplatin and calculated the IC50 of carboplatin to OC organoids. The results showed that organoids could be stably passaged *in vitro*, and patients receiving neoadjuvant chemotherapy had higher resistance to carboplatin (Zhang L. et al., 2023). Bose et al. constructed ascitic organoids derived from advanced and/or recurrent high-grade SOC patients and transfected HyPer DAAO to express them. The study found that the HyPer signal in PDOs of carboplatin-resistant patients was significantly higher than that of carboplatin-sensitive patients (Bose et al., 2022). Witte et al. found that organoids can maintain the genomic characteristics of the original tumor lesion, reflecting the patient’s response to neoadjuvant carboplatin or paclitaxel combination therapy. PDO shows heterogeneity in drug response to chemotherapy and targeted drugs between and within patients. Using patient-derived organoids for *in vitro* drug screening, 88% of patients were found to have high reactivity to at least one drug through drug screening (de Witte et al., 2020). Sun et al. established organoids from cisplatin sensitive and resistant OC tissue samples and found that serine/threonine kinase (Aurora-A) induces cisplatin resistance by regulating cell aging and glucose metabolism through involvement in SOX 8/FOXK 1 signaling in OC (Sun et al., 2020).

Ito et al. performed sensitivity testing on paclitaxel and carboplatin using organoids prepared from cancer tissue primary spheroids (CTOS) of OC patients undergoing chemotherapy, with a success rate of 84% (Ito et al., 2023). Extensive sensitivity was observed in organoids of both drugs. Of the 18 patients in whom clinical response information was available, all four clinically resistant organoids showed resistance to both drugs. Among the 18 cases, five had dual drug resistance, and their response rate was consistent with the clinical response rate. It was also found that several drugs combined with carboplatin had better effects than paclitaxel, and some drugs such as afatinib showed cumulative effects with carboplatin (Ito et al., 2023).

Taken together, the above examples indicate that OC organoids derived from patients have a clinical translational role in predicting drug response and resistance mechanisms and play a major role in precision medicine.

## 3 Endometrial cancer (EC) organoids

The incidence rate and mortality of EC in developed countries have increased significantly every year (Sung et al., 2021). The treatment options for women with advanced EC are limited, and the currently available treatment methods are not ideal. Developing effective and precise targeted therapies requires a real preclinical model, and the organoid model of EC is of great significance for the pathological status of EC.

### 3.1 Disease modeling

At present, there is no mention of specific histological types for modeling EC organoids. However, by obtaining organoids from patient tissues and constructing EC organoid models, it is helpful for the research of EC. The following experiments and studies show the modeling method for EC organoids in the study.

The matrix gel method is the main culture method for EC-like organs, with the main difference being the tissue source. Directly obtaining tissue samples from EC tissue and then culturing them into EC-like organs is a common method. For example, the EC tissue collected by Xue et al. was subjected to *in vitro* treatment and culture after excision, and organoids were cultured using the matrix gel method (Yang et al., 2023). The specific method is to embed a single cell or organ-like fragment into the ECM of Engelbreth-Holm-Swarm (EHS) mouse sarcoma and distribute it to the surface of the plastic container for warm tissue culture in the form of small droplets. After the ECM is incubated at 37°C, it will solidify into a gel, which can then be covered with the culture medium (Yang et al., 2023). Organoid will develop into 3D structure in the dome. Jamaluddin et al. constructed organoids from tissue biopsies collected from four patients with EC and cultured them using the matrix gel method. They found that proteomic differences observed in the same tumor in patients were also transformed into differences in tumor cell growth rate (Jamaluddin et al., 2022). Su et al. cultured fresh tumor tissues from patients with EC using the matrix gel method and analyzed these organoids with different estrogen-related receptors α (ERR α) Sensitivity of organoids to DDP at expression levels. Studies have shown that the proportion of organoid fragments significantly increases in a dose-dependent manner after cisplatin treatment, with estrogen related receptors α (ERR α) The inhibition promotes the activation of NLRP3/caspase1/GSDMD pyroptosis pathway in EC cells (Su et al., 2023). Berg et al. obtained fresh tumor tissue from patients with malignant endometrial diseases and established EC organoids by using the matrix gel method. They found that removing N2 supplementation and adding ROCK inhibitors can enable long-term expansion and cryopreservation of organoids, which can reflect the genetic characteristics of endometrial tumors and predict patient prognosis through organoid models (Berg et al., 2021).

Some studies have explored new sources of organization and cultivation methods. For example, Katcher et al. collected endometrial samples from patients and used the matrix gel method to establish cancerous and normal endometrial tissue organoids for further analysis for DNA and RNA extraction and histological analysis (Katcher et al., 2023). Hsin et al. used xenografts (PDX) from patients with EC to construct organoids. They subcutaneously implanted tumor samples from EC patients into late stage severely immunodeficient mice, killed the mice, and conducted organoid formation experiments and primary cell culture via the matrix gel method (Hsin et al., 2023). Their study used repeated up and down pipetting to dissolve organoids for passage (Hsin et al., 2023). Sengal et al. collected PDX tumors from EC patients, dissected, and placed some of the tumors in RPMI culture medium containing antibiotics (penicillin/streptomycin) and antifungal drugs, and constructed EC organoids using the matrix gel method (Sengal et al., 2023). Maru et al. used a matrix gel bilayer organoid culture scheme to construct organoids to explore whether the combination of Kirsten rat sarcoma viral oncogene homolog (*Kras*) gene activation and *Pten* inactivation can transform mouse endometrial organoids in the subcutaneous tissue of immunodeficient mice. This study found that in endometrial organoids expressing *Kras* (G12D), *Pten* knockdown does not confer tumorigenicity, but *Cdkn2A* knockdown or *Trp53* deficiency leads to the development of carcinosarcoma (CS). The carcinogenic potential of *Kras* (G12D) and the histological characteristics of derived tumors depend on the environment and vary depending on organ type and the experimental environment (Maru et al., 2021a).

In summary, the main method for modeling EC-like organs is to isolate tumor cells from EC tissue (from surgery, biopsy, xenografts from EC patients, and EC mice); use matrix gel method; chop; rinse; digest; and filter; and embed them into a 3D matrix, and cultivate them in a culture medium supplemented with various growth factors and hormones. However, the specific experimental conditions reported in each study are different and have been specifically listed above. Wu et al. optimized the current EC like organs by introducing cancer-associated fibroblasts (CAFs) isolated from EC lesions (Wu et al., 2022). Based on co-cultivation of CAFs and EC-like organs, they found that CAFs can promote the growth of EC-like organs, possibly by secreting factors. According to the results, CAFs can also promote growth, providing a more promising model for the basic and preclinical research of EC (Wu et al., 2022).

### 3.2 Drug efficacy evaluation and drug response prediction

Similar to OC organoids, to better simulate the treatment response of tumors, existing studies have constructed EC organoid models, retaining the histological and genetic characteristics of the original tumor as well as tumor heterogeneity to evaluate drug efficacy and predict drug response.

#### 3.2.1 Drug efficacy evaluation

Xue et al. evaluated the effectiveness of the small molecule inhibitor SMIP34, which inhibits PELP1 oncogenic signaling, in the treatment of EC. SMIP34 was used to treat *in vitro* patient-derived explant-like organs and cells, and it was found to significantly reduce cell viability, colony forming ability, and the ability to induce apoptosis (Yang et al., 2023). Spencer et al. evaluated the drug efficacy of LIFR inhibitor EC359 by constructing EC organoids. The organoid viability analysis established through primary type II EC tissue showed that compared with dose-dependent vector therapy, the new small molecule LIFR inhibitor EC359 treatment reduced its viability and inhibited the growth of EC organoids, indicating that LIFR inhibitor EC359 may be a new small molecule therapy for the treatment of type II EC (Spencer et al., 2023). Hsin et al. validated using organoids and primary cells derived from xenografts (PDX) from EC patients β-catenin inhibitory ability of the catenin inhibitor ICG-001 on EC was studied, and the results showed that ICG-001 can inhibit PDX-derived organoids and primary cells (Hsin et al., 2023).

By constructing an EC organoid model to test the drug efficacy of antisense oligonucleotides (ASO), a study found that ASO targeting SNORD14E inhibited the growth of EC (Chen et al., 2023). Chen et al. cultured the mouse EC model with *Trp53*, *Pten*, and *Pik3r1* mutations and overexpression of *Myc* and *Kras* (G12D) in the medium containing matrix gel, established EC-like organs by matrix gel method, screened therapeutic drugs for EC, and found that the menin-MLL inhibitor affects the progress of EC by regulating the HIF pathway. MI-136 significantly inhibits the growth of EC-like organs from patients. The results showed that MI-136 can serve as a potential inhibitor of EC by regulating the HIF pathway (Chen et al., 2021).

#### 3.2.2 Drug reaction prediction

Sengal et al. tested the drug sensitivity of Fibroclast Growth Factor Receiver (FGFR) through the establishment of EC organoids, indicating that PDX-derived organoids and PDX with FGFR 2c subtype expression are sensitive to FGFR inhibition (Sengal et al., 2023). Berg et al. cultured the tumor tissue of excised EC patients into organoids and amplified it in a culture medium determined by chemical composition to predict patient prognosis and provide more effective medication. The OEC-07-G3 cell line was found to be highly sensitive to carboplatin–paclitaxel, and the survival rate measured after combination treatment with carboplatin (200 µM) paclitaxel (200 nM) was only 5.4% (Berg et al., 2021).

All the above examples indicate that EC-like organs derived from patients have a clinical translational role in predicting drug response and resistance mechanisms and play a major role in precision medicine.

## 4 Cervical cancer (CC) organoids

Cervical cancer is the fourth-most commonly diagnosed and lethal cancer among women (Sung et al., 2021; Zhang et al., 2021). The vaccination rate and cervical screening rate of human papillomavirus (HPV) in low- and middle-income countries are still quite low (Doherty et al., 2016; Spayne and Hesketh, 2021). For decades, this has exacerbated the ongoing burden of CC in developing countries. The treatment options for CC, including surgical resection, radiotherapy, and chemotherapy, have limited efficacy and may have significant toxicity to patients with recurrence or metastasis (Chung et al., 2019). In recent years, immunotherapy has become a promising method for treating CC (Hu and Ma, 2018). However, due to the complexity and heterogeneity of solid tumors, the efficacy of immunotherapy varies among patients (Hegde and Chen, 2020; Ando et al., 2021). Therefore, it is necessary to develop preclinical models that can accurately evaluate the efficacy and mechanisms of these therapies.

### 4.1 Modeling of CC organoids

At present, cervical small-cell neuroendocrine carcinoma (SCNEC) organoids (Masuda et al., 2023), cervical small-cell carcinoma (SCCC) organoids (Kusakabe et al., 2023), cervical clear-cell carcinoma (cCCC) organoids (Maru et al., 2019a), and squamous cell carcinoma and adenocarcinoma (AdCA) organoids (Lõhmussaar et al., 2021) have been established. By constructing an organoid model of CC in patients, preserving the genomic map of the primary tumor, and identifying the genetic correlations related to the progression of CC can contribute to the study of disease etiology. The lack of a human derived *in vitro* model that can summarize cervical precancerous lesions has always been a bottleneck in the study of HPV infection-related precancerous lesions and cancer. By constructing an organoid model covering patient sources of HPV-related cervical precancerous lesions and cancer, preserving genomic and transcriptomic features, as well as pathogenic HPV genome, an experimental platform and biological library have been established for the *in vitro* mechanism research, therapeutic vaccine screening, and personalized treatment of HPV-related cervical diseases (Kusakabe et al., 2023; Hu et al., 2024).

Masuda et al. used the matrix gel method to prepare SCNEC organoids from patient tumors or mouse xenografts. Histologically, organoids and xenograft tumors showed clear differentiation into SCNEC or AdCA in certain areas and unclear differentiation in some areas (Masuda et al., 2023). By tracking single cells, the existence of cells with dual potential differentiation towards SCNEC and AdCA was revealed. Single-cell transcriptome analysis identified three distinct clusters: SCNEC-like clusters, AdCA-like clusters, and clusters lacking specific differentiation markers. The expression of neuroendocrine markers is enriched in SCNEC-like clusters, but not completely enriched. HPV 18 E6 is enriched in SCNEC-like clusters, exhibiting higher proliferation and lower levels of p53 pathway. After anti-cancer drug treatment, the expression of AdCA markers is increased, whereas the expression of SCNEC is decreased. The report system using keratin 19 expression revealed that the changes in cell differentiation were related to the differentiation transformation induced by drug therapy. These data indicate that mixed SCNEC/cervical tumors have a clonal origin, characterized by unclear differentiation status (Masuda et al., 2023).

Kusakabe et al. obtained surgical specimens from a patient diagnosed with HPV18-positive SCCC and performed organoid culture using the matrix gel method (Kusakabe et al., 2023). The results of HPV18-positive SCCC organoids culture and drug sensitivity tests using mouse xenograft models derived from the organoids showed that KRAS pathway inhibitors had stronger anti-cancer effects on SCCC organoids than the Myc inhibitors, which was also confirmed in the xenograft models (Kusakabe et al., 2023).

Maru et al. successfully established the first cCCC organoid using the matrix gel method, demonstrating the sensitivity of cCCC to major chemotherapy drugs and MET inhibitors. This indicates that the organoid derived from the tumor retains the morphology and genetic abnormalities of the original tumor, providing a reference for the treatment of cCCC (Maru et al., 2019a). Lõhmussaar et al. collected materials from Pap smears of patients with squamous cell carcinoma (SCCa) and AdCa CC and established CC-like organs using the matrix gel method to study cervical histological dynamics. After successfully establishing a 3D CC-like organ, personalized medical methods for CC were studied (Lõhmussaar et al., 2021). Hu et al. established a long-term 3D organoid culture protocol using the matrix gel method, and established an organoid model covering patient sources of HPV-related cervical precancerous lesions and their cancers. The model retained genomic and transcriptomic characteristics as well as pathogenic HPV genome. This study was the first to establish a cervical precancerous lesion model containing HPV, providing an experimental platform and biological library for *in vitro* mechanism research, therapeutic vaccine screening, and personalized treatment of HPV-related cervical diseases (Hu et al., 2024).

Toyohara et al. constructed CC organoids using the matrix gel method, transfected the lentiviral vector (HPV18LCR-GFP vector) into squamous columnar junction (SCJ) organoids from patients, and evaluated the presence of green fluorescence protein (GFP)-positive cells (Toyohara et al., 2023). The results showed that pathways related to cell cycle and viral carcinogenesis were upregulated in GFP-positive cells, while keratinization and mitochondrial autophagy/autophagy-related pathways were upregulated in GFP-negative cells. Among the upregulated genes, *ADNP*, *FHL2*, and *NPM3* were significantly associated with the activation of the early promoter of HPV18 and the maintenance of the HPV18 gene group. Thus, the initial replication mechanism of HPV18 and the breakthrough in the origin of HPV18-related CC cells were determined (Toyohara et al., 2023).

In summary, the main method for modeling CC organoids is by constructing organoids using the matrix gel method by isolating tumor cells from CC tissue (obtained by surgery, biopsy, smear, patient tumor, or mouse xenograft). The key to successful organoid culture is digestion of the original tissue and composition of the culture system. During the digestion process of the original tissue, it is necessary to increase the number of cells as much as possible while ensuring cell viability. Therefore, Hu et al. constructed an organoid model covering patient sources of HPV-related cervical precancerous lesions and their cancers, and innovatively used an enzyme mixture called SIL tissue dissociation solution to obtain a sufficient number of cells to the maximum extent possible (Hu et al., 2024). In addition, the tissue obtained from constructing organoids varies. Toyohara et al. constructed organoids by obtaining normal tissue from the squamous columnar junction area of patients undergoing a total hysterectomy. The specific experimental conditions reported in each study of organoids vary, as detailed in the above sections (Toyohara et al., 2023).

### 4.2 Drug testing and screening

The application of organoids in promoting precision medicine, drug detection, and screening is rapidly expanding (Kopper et al., 2019; Shi et al., 2020). To better simulate the treatment response of tumors, organoids were constructed using tissues from different patient sources, and different CC disease models were constructed. The histological and genetic characteristics of the original tumor as well as tumor heterogeneity were preserved, and drug efficacy evaluation or drug sensitivity testing and drug screening experiments were carried out (Lin et al., 2023a; Hu et al., 2023).

Hu et al. explored the novelty of the therapeutic effect of synchronous radiotherapy and chemotherapy on CESC by constructing organoids for the same (Hu et al., 2023). The research results showed that high expression of Lumican (LUM) affected the immune microenvironment of organoids in patients with CESC treated with synchronous radiotherapy and chemotherapy. High expression of LUM was associated with poor efficacy in CESC patients receiving synchronous radiotherapy and chemotherapy, possibly by affecting the PAR and IL1 signaling pathways of the immune landscape (Hu et al., 2023). Lin et al. explored the sensitivity of drug action by constructing CC-derived organoids and found a significant heterogeneity in carcinogenic and tumor microenvironment between CSCC and CAde pathological types. CAde has a more inhibitory immune microenvironment, and lapatinib (an ERBB2 inhibitor) is particularly sensitive to CAde samples. Dasatinib and Doramamod targeting STAT5 and MAPK molecules exhibit specific sensitivity to CSCC cancer cell lines and organoids (Lin et al., 2023a).

Fonte et al. explored the therapeutic effect of the combination of trabectedin and propranolol by constructing organoids. Research has shown that trabectedin can reduce the proliferation of organoid cell lines derived from CC patients, and trabectedin is associated with β-blocker propranolol combined therapy to counteract the effects in CC models β- Activation of adrenergic receptors leads to resistance to trabectedin (Di Fonte et al., 2023). Lõhmussaar et al. established CC organoids in SqCa and AdCa and evaluated possible p53 pathway defects in organoids using the p53-activating compound, Nutlin-3a (Lõhmussaar et al., 2021). According to genomic data, the p53 mutant SqCa-1.2 line is the most resistant, and the SqCa-3 line shows the highest resistance to treatment with two platinum analogues, while the SqCa-6 and SqCa-7 lines are highly sensitive to gemcitabine (Lõhmussaar et al., 2021). The PDO biobank established by Huang et al. contains 67 cases of heterogeneous CC organoids, and their *in vitro* responses indicate that they can capture the radiation heterogeneity of patients (Huang et al., 2023). To simulate an individual’s response to adoptive T cell therapy (ACT), tumor infiltrating lymphocytes (TILs) were amplified *in vitro* and co-cultured with paired organoids. The PDOs TILs co-culture system showed significant response, supporting the potential of the PDOs platform in guiding prospective intervention trials for CC treatment (Huang et al., 2023).

All the above examples indicate that CC organoids derived from patients have a clinical translational role in drug screening and predicting drug reactions and resistance mechanisms and play a major role in precision medicine.

**FIGURE 1:**
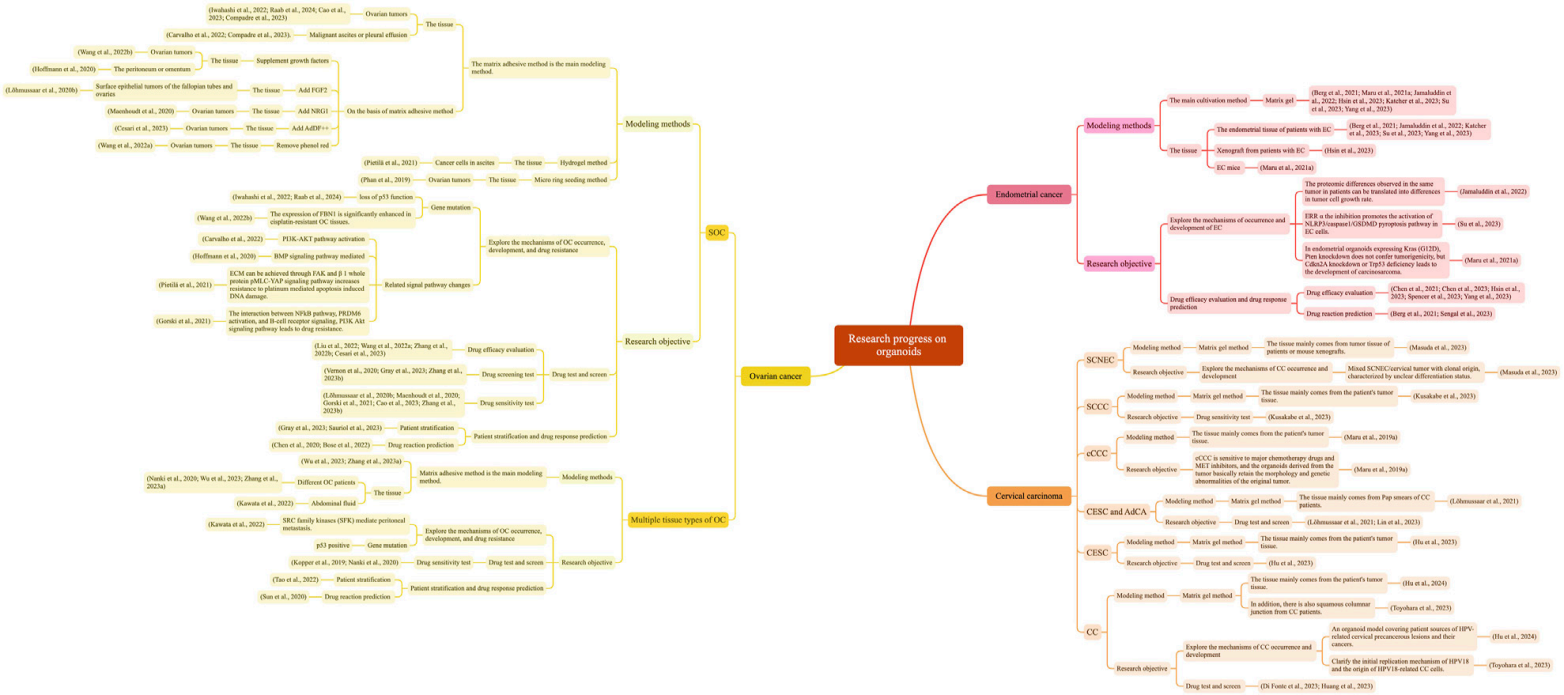
Research progress on organoids of OC, EC, and CC with Xmind.

We have summarized the research progress of OC, EC, and CC organoids in Figure 1.

## 5 Discussion

### 5.1 Organoids and the three major gynaecological malignant tumors

#### 5.1.1 Main modeling methods, application fields, and future directions of OC organoids

In terms of disease modeling, various histological subtypes of organoids have been successfully developed, including SOC organoids, clear cell carcinoma, endometrioid OC, and epithelial OC organoids. Based on the above summary of modeling methods for OC organoids, we found that the main method for modeling OC organoids is to construct organoids using the matrix gel method by isolating tumor cells from OC tissue (from surgery, biopsy, ascites or pleural effusion, high-purity tumor deposits in the peritoneum/omentum). However, the specific experimental conditions reported in each study vary; for example, some studies have adopted improved methods by adding advanced DMEM/F12 medium containing specific growth factors and niacinamide (Willert et al., 2003). In addition, some studies have used the hydrogel method to construct OC-like organs (Pietilä et al., 2021). Phan et al. found that this cultivation method not only did not affect the growth of organoids but also afforded automated high-throughput screening of chemotherapy drugs when using the micro ring seeding method (Phan et al., 2019). When multiple tissue types of organoids are cultivated simultaneously, using a cocktail medium to cultivate organoids can achieve a total success rate of 80% (Nanki et al., 2020). In addition, the use of Accumax 7-min enzymatic hydrolysis increased the success rate of organoid proliferation in tumors of different stages and subtypes from 45% to 90% (Maru et al., 2019b). In terms of medium additives, Hoffmann and Lõhmussaar et al. found that epidermal growth factor (EGF) is an essential component, and the addition of neuromodulatory protein-1 (NRG1) is the optimal key factor for the development and growth of OC-like organs (Lõhmussaar et al., 2020b; Hoffmann et al., 2020; Maenhoudt et al., 2020). In addition, in the extraction of OC organoids, establishing organoids derived from omental histology facilitates the exploration of relevant metabolic mechanisms (Lõhmussaar et al., 2020b). By constructing a model of organoid diseases and constructing OC models with different gene expressions, we aimed to explore the relationship between genes and the occurrence, development, and prognosis of OC (Iwahashi et al., 2022; Raab et al., 2024); identify ligands and receptors associated with poor survival in OC (Carvalho et al., 2022); study chemotherapy resistance mechanisms (Gorski et al., 2021); explore platinum-based chemotherapy response and survival markers for OC (Wang Z. et al., 2022; Compadre et al., 2023); and explore the role of signaling pathways in OC (Hoffmann et al., 2020). In terms of drug detection and screening, by constructing an OC organoid model and summarizing the characteristics of the tumor, drug screening experiments (Nanki et al., 2020; Vernon et al., 2020; Wang W. et al., 2022; Zhang et al., 2022a, 2022b; Liu et al., 2022; Cesari et al., 2023; Wan et al., 2023) and drug sensitivity tests (Kopper et al., 2019; Lõhmussaar et al., 2020b; Maenhoudt et al., 2020) can be conducted to explore the mechanisms of drug resistance and drug action in OC (Zhang X. et al., 2023; Cao et al., 2023). Meanwhile, molecular heterogeneity exists in OC, leading to differences in drug responses among different patients. Organs that retain the heterogeneity and genetic characteristics of the original tumor have an advantage in precision medicine. *In vitro* drug testing of OC organoids from patient sources is a new method in precision medicine (Chen et al., 2020; Zhang L. et al., 2023; Gray et al., 2023; Sauriol et al., 2023).

#### 5.1.2 Main modeling methods, application fields, and future directions of EC organoids

In terms of disease modeling, the currently developed EC organoid modeling can be stored for a long time, and can be used for downstream histological and genomic characterization as well as functional determination, such as evaluating response to therapeutic drugs (Katcher et al., 2023). Moreover, the proteomic differences in organoid tissues are consistent with the growth rate of tumor cells (Jamaluddin et al., 2022). Therefore, organoids that can highly preserve the structure and function of primary tissues have broad prospects in exploring the mechanisms driving tumor development (Maru et al., 2021a; Sahoo et al., 2022). At present, the main method for modeling EC organoids is to isolate tumor cells from EC tissue and use the matrix gel method to construct organoids. However, different specific methods have not been studied. Most studies focus on EC patients, but some studies have constructed organoids by collecting EC mouse tissue (Sahoo et al., 2022), and some others have transformed mouse endometrial organoids into subcutaneous tissue (Maru et al., 2021a). Another study has found that removing N2 from the culture medium and adding ROCK inhibitors can promote long-term expansion and cryopreservation of organoids (Berg et al., 2021). Wu et al. optimized the current EC-like organs by introducing CAFs isolated from EC lesions (Wu et al., 2022). Based on co-cultivation of CAFs and EC-like organs, they found that CAFs can promote the growth of EC-like organs, possibly by secreting factors. According to the results, CAFs can also promote growth, providing a more promising model for the basic and preclinical research of EC (Wu et al., 2022). In addition, EC organoids can be used for drug detection and screening (Chen et al., 2023; Hsin et al., 2023; Spencer et al., 2023; Su et al., 2023; Yang et al., 2023) and drug screening (Chen et al., 2021) to explore the efficacy and mechanism of drug action. In addition, by constructing organoids from patient sources, it is possible to accurately predict the effects of drug action on different patients, playing a role in precision medicine (Berg et al., 2021; Sengal et al., 2023).

#### 5.1.3 Main modeling methods, application fields, and future directions of CC organoids

Published studies have reported the establishment of SCNEC organoids, SCCC organoids, cervical transparent cell carcinoma organoids, CESC organoids, and AdCA organoids, constructed CC organoids, captured the characteristics of histological cancer subtypes, and constructed organoid models covering patient sources of HPV-related cervical precancerous lesions and their cancers. Studies have also established the experimental platforms and biobanks for *in vitro* mechanism research, therapeutic vaccine screening, and personalized treatment of HPV-related cervical diseases (Kusakabe et al., 2023; Hu et al., 2024). At present, the main method for modeling CC organoids is to isolate tumor cells from CC tissue. In addition, some studies have also constructed organoids using the matrix gel method by using normal squamous columnar junction tissue from patients undergoing total hysterectomy (Toyohara et al., 2023). In addition, studies have shown that culture media play an important role in constructing organoids of different tissue types (Huang et al., 2023). The most important key to successful organoid culture is the digestion of the original tissue and the composition of the culture system. During the digestion process of the original tissue, it is necessary to increase the number of cells as much as possible while ensuring cell viability. Therefore, studies have also attempted to improve the treatment of CC tissue with tissue dissociation solution to obtain a sufficient number of cells to the maximum extent (Hu et al., 2024). Moreover, CC organoids can also be used for the detection of different drugs (Di Fonte et al., 2023; Hu et al., 2023) and sensitivity testing (Lin et al., 2023a). Similar to OC and EC, CC organoids that highly preserve the heterogeneity of the original tissue can be used for personalized and precise treatment, exploring drugs for experimental treatment in patients with different gene mutations (Lõhmussaar et al., 2021; Huang et al., 2023).

### 5.2 Organoid modeling application insights

#### 5.2.1 Limitations of existing tumor models

Gynecological malignant tumors are major diseases that threaten women’s lives, and traditional treatments fail to ensure the efficient killing of tumor cells by drugs and a good prognosis after treatment, mainly because the tumor and its microenvironment are not well understood. Therefore, it is crucial to find a suitable model that is stable and representative of the complex structure of human tumor tissues and other tissues. Popular tumor models in recent years mainly include traditional cell line models and xenograft models. However, these models have limitations, perhaps not replicating well the tissue complexity and genetic heterogeneity of tumors, poorly reproducing the clinical response of patients, and failing to predict clinical response.

Traditional cell line models mainly refer to 2D cell line models. 2D cell cultures lack the ability to regulate cell behavior, cannot be modeled based on cell-cell/cell-ECM interactions, and often adhere to the surface of the plastic medium to form a monolayer of cells, which are directly and uniformly exposed to intra-medium factors that fail to replicate the actual microenvironment of the tissues, whilst the 2D *in vitro* culture conditions require extensive selection and adaptation, and since only rare clones are able to expand and maintain multiple passages, derived cell lines may have undergone substantial genetic changes that make it difficult to reproduce the genetic heterogeneity of the original tumor (Li and Zhang, 2023; Cheng et al., 2024). Peng D. et al. developed CancerCellNet technology based on transcriptomic analysis for assessing the match between different cancer models and primary tumors *in vivo*, and showed that human cancer cell lines are not genetically identical to cancer cells *in vivo*, suggesting that highly transmissible cell lines sometimes undergo potential evolution, and that the 2D cell line models do not accurately represent the genotypic and pathological characteristics of the primary tumor cells (Peng et al., 2021).

Xenograft models are able to mimic human tumor biology, although the limitations are limited efficiency and the high cost of transplantation for subpopulations of patient tumors (Li and Zhang, 2023). Yuan Z. et al. analyzed unlocalized RNA-Seq reads from 184 experiments to assess the extent of viral infection and its effect on patient-derived xenografts, and found that a certain anticancer drug turned out to appear to kill tumor cells, when in fact, because human tumors are infected by mouse viruses when implanted in mice, many anti-cancer drugs that kill tumor cells in mouse models do not work in human trials (Yuan et al., 2021).

#### 5.2.2 Characteristics of organoid modeling applications

Organ-like structures are simplified or formed in 3D culture systems and can reproduce the structure and physiology of most female reproductive tissues (Kessler et al., 2015; Turco et al., 2017). Experiments on organs provide a unique opportunity, as they are translatable, repeatable, and scalable. They are produced from pluripotent stem cells or adult stem cells, making them a special 3D culture system that can closely mimic the structure and physiology of the originating tissue (Kim et al., 2020). Given the high heterogeneity of gynecological tumors, organoids can highly reproduce the original characteristics of tumors *in vivo* and *in vitro*, and have broad application potential in disease mechanism research, drug research, predicting patient response to treatment, and providing personalized medical solutions for patients (Liu et al., 2020). Gynecological cancer organoids are widely used as the best model for studying tumor biology (Salinas-Vera et al., 2022). This is because using this model system enables scientists to better understand the development mechanism of tumors, disease progression, and drug efficacy, thereby providing patients with more reliable treatment options. These studies are of great significance for discovering new anti-cancer drugs and improving patient survival rates. Overall, the application of gynecological cancer organoids in the field of tumor biology has played a crucial role in the development of clinical medicine. Moreover, gynecological tumors are more complex, and different patients may have significant differences in their response to the same clinical treatment. For gynecological tumors with extremely high malignancy, the mechanisms of tumor progression, drug efficacy, and drug resistance remain unclear (Tendulkar and Dodamani, 2021). A large number of anti-cancer drugs that have passed Phase-I drug safety tests have been eliminated in Phase-II and III efficacy tests, while patient-derived tumor organoids (PDTO) can optimize preclinical efficacy in models to improve the prediction of clinical treatment responses and reduce the failure rate of clinical trials, thus solving this important problem (Jin et al., 2020). Gynecological patients often exhibit resistance to chemotherapy and radiation therapy, and traditional treatment methods may not be effective in inhibiting tumor growth and metastasis. Some patients may even receive overly toxic treatments without significant results (Kopper et al., 2019). By using organoid culture models, researchers can predict the patient’s response to different treatment methods, thereby tailoring specific treatment plans for each patient. Through personalized treatment, doctors can more accurately select drugs and doses that are suitable for patients and adjust them according to the patient’s condition at any time. In this way, patients can achieve better treatment outcomes without having to endure the side effects of excessively toxic drugs, thereby achieving precise treatment. In addition, most gynecological tumors progress rapidly, and patients are often diagnosed in the middle and late stages. Hence, it is particularly important to develop a suitable treatment plan. We can predict the response of patients to biopsy tissue treatment through organoids to select the most suitable treatment plan for patients (Lõhmussaar et al., 2020a).

We should acknowledge the limitations associated with organoid-based treatments, such as a lack of scalable organoid lineages with the ECM and immune components, and a lack of interaction with native ECM components (Francés-Herrero et al., 2022). Organs exhibit relatively random growth properties, do not support interorgan communication, and lack vascular systems and immune cells (Shankaran et al., 2021). The random distribution of organoids in the matrix gel can cause differences in spatial density and spacing, leading to uncontrolled variations in organoid phenotype. The variability related to patient tissue origin and culture treatment still needs to be further optimized. In addition, the differences in matrix gel (including differences in physical properties and growth factor content) can directly affect the stability of organoid cultures, and frequent manual operations during the cultivation process can introduce human errors, thereby affecting the stability of the culture system. Therefore, to improve the success rate of organoid generation and research, it is crucial to develop standardized protocols for routine organoid-based treatment (Clinton and McWilliams-Koeppen, 2019). The current results show that the synthetic guiding hydrogel can closely imitate the structure and characteristics of the natural ECM in cancer tissue and has appropriate clues to support tumor growth, migration and organoid invasion *in vitro*, so it has a potential in future organoid culture research (Jia et al., 2022).

#### 5.2.3 Prospects for the application of organoid modeling

##### 5.2.3.1 Range of diseases

In summary, we found that these three types of organs have gradually developed to the stage of technological maturity through a review of OC-like organs, EC-like organs, and CC-like organs. At the same time, more types of gynecological tumor-like organs are also being developed. Lõhmussaar et al. established an *in vitro* model of fallopian tube and ovarian surface epithelial cell (OSE) tumor development by targeting the mouse *Trp53* gene (Lõhmussaar et al., 2020b). Mouse fallopian tubes and OSE tissues were dissected, subjected to different enzyme treatments, embedded in basement membrane extract (BME), and cultured in appropriate media to ultimately cultivate fallopian tube- and ovarian tumor-like organs (Lõhmussaar et al., 2020b). Subsequently, Maru et al. utilized mouse fallopian tube organoids to investigate the tumorigenic potential of recombinant gene interactions (Maru et al., 2021b). The results showed that inhibition of *Pten* and simultaneous induction of *Pik3ca* mutation led to the development of *in situ* cancer and high-grade serous tumors, respectively, reflecting the frequent activation of the PI3K/AKT axis and the impact of activation of the Wnt pathway in HGSC on tumor generation (Maru et al., 2021b). Meanwhile, studies have also established a mouse model of fallopian tube organoids and found that the ALDH1A family inhibitor (ALDHi) 673A reduced organoid complexity and significantly reduced colony formation in *BRCA* mutant cells, suggesting that ALDHi 673A can serve as a chemopreventive agent for *BRCA1/2* mutation carriers (McGonigal et al., 2023). Yang et al. established cancerous organoids in the ascites of OC patients and found that miR-1246 and miR-1290 shuttle through malignant ascites-derived extracellular vesicle (EVs) by regulating the common target ROR α to promote the invasion and migration of OC cells (Yang et al., 2022). Recently, Diaz et al. generated patient-derived organoids from liquid biopsies of patients with gynecological serous carcinoma (GSC) and found that the histological and immunofluorescent characteristics of ascites-derived organoids were similar to those of corresponding primitive tumors, and the evaluation of platinum sensitivity in these preclinical models replicated the clinical environment of corresponding GSC patients (Arias-Diaz et al., 2023). The study showed that cell response to DNA damage stimulation is the main biological process associated with obtaining resistance to first-line treatment for GSC [100]. It can be seen that the organoid culture model in gynecology provides a valuable platform for studying the molecular processes that lead to uncontrolled cell proliferation and metastasis. This model can simulate the tumor microenvironment in the human body in more detail, including the interaction between tumor cells and surrounding tissues, thereby providing more accurate research results. Gynecological organoid culture models have higher biological reliability than traditional cell culture models (Francés-Herrero et al., 2022). This means that researchers can better understand how tumor cells grow and spread and study the molecular mechanisms that lead to this uncontrolled process. By gaining a deeper understanding of these mechanisms, scientists can identify new drug targets and develop more effective treatment strategies.

##### 5.2.3.2 Research directions

As an emerging *in vitro* 3D modeling technology, organoid models reproduce the physiological and pathophysiological characteristics of the original tissues *in vivo* and are widely used in a variety of fields such as drug discovery, disease modeling, cancer research, developmental biology, regenerative mechanisms, precision medicine and organ transplantation. Tumors are not only aggregates of malignant cells but also well-organized complex ecosystems. The immune component within the tumor, called the tumor immune microenvironment, has long been shown to be closely associated with tumor development, recurrence, and metastasis (Fu et al., 2021). Therefore, more scholars have also initiated studies on the role of organoids in tumor immunotherapy.

Organoids can be co-cultured with immune cells. Zhou G. et al. established immune organoids by co-culture protocol of cholangiocarcinoma organoids and peripheral blood T cells and successfully mimicked effective anti-tumour immune response *in vitro* (Zhou et al., 2022). Chan I. S. et al. co-cultured exogenous immune cells directly with tumor epithelial cells premixed in matrix gel to simulate the interaction between immune cells and tumor cells without the need to establish tumor-like organs in advance (Chan and Ewald, 2022). In addition, organoid and tumor immunity can still be studied using air-liquid interface (ALI), and 3D microfluidics (Zheng et al., 2019; Esser et al., 2020).

Organoids have great potential for tumor immunotherapy development. TCR sequencing of peripheral blood T cells from pancreatic cancer organoid co-cultures revealed significant expansion of a specific subpopulation of T cell clones, while a model constructed from a mixture of organoids and cognate immune cells could also be used to induce peripheral blood T cell killing of tumor cells, demonstrating that the model is a viable platform allowing individual patient immune systems and tumor cells to be investigated in the context of an individualized immunotherapeutic responses while maintaining viability (Lin et al., 2023b). Jacob F. et al. report the generation and biopreservation of patient-derived glioblastoma-like organoids (GBOs) by co-culturing glioblastoma-like organoids with CAR-T cells, demonstrating the ability of CAR-T cells to specifically kill the target cells instead of completely eliminating all tumor cells, and that this co-culture model provides a viable approach to testing the efficacy of CAR-T therapies (Jacob et al., 2020).

The emergence and continuous development of organoids have become a new technology in anti-tumour immunotherapy research, with great potential for mimicking the effects of immunotherapy, investigating drug resistance mechanisms, and developing new combination therapies, which means that cutting-edge researchers can devote their attention to organoid immunotherapy as a point of intervention to promote the clinical translation of tumor immunotherapy, and ultimately help to achieve personalized immunotherapy.

##### 5.2.3.3 Models of training

Owing to the rapid progression of most gynecological tumors and the fact that patients are often diagnosed in the middle and late stages, it is particularly important to develop an appropriate treatment plan. We can predict the response of patients to biopsy tissue treatment through organoids to select the most suitable treatment plan for patients (Lõhmussaar et al., 2020a). However, traditional organoid culture systems rely on manual operations, making the cultivation process more cumbersome. Additionally, individual and batch differences in culture also limit the transformation and application of organoids (Corrò et al., 2020). Therefore, we also need to find rapid methods for cultivating organoids to enable patients to receive personalized treatment as soon as possible. The emergence of organoids provides new development opportunities for research in fields such as disease modeling, drug screening, and tissue development (Rossi et al., 2018). The human organ chip simulates the structure, function, and microenvironment of human tissues or organs by constructing miniature tissues or organs on microfluidic chips. Therefore, using microfluidic chip technology to construct a high-throughput automated organoid culture system can greatly improve cultivation efficiency, reduce human errors and batch differences, and accelerate organoid transformation and application (Shirure et al., 2021). At present, according to the different target organs constructed, organ chips can be divided into lung chips (Kim et al., 2024), brain chips (Wang et al., 2018; Song et al., 2022), kidney chips (Lee et al., 2021), and intestinal chips (Hakuno et al., 2022) for drug screening and safety evaluation. Therefore, with the advent of the digital era, the combination of gynecological tumor organoids and organ chips has become an important trend. The in-depth application of this technology in the field of gynecological tumors provides a high-quality platform for disease mechanism research, clinical diagnosis, and target drug discovery.

### 5.3 Limitations of the current study

Currently, there are still many issues facing research on gynecological malignancies and organoid studies. 1) Research is currently focused on different studies of organoid models, and although the research aspects show diversity, the experimental results lack reproducibility due to biological experimental materials, laboratories, researchers, and other factors. 2) The type of methodological research is more focused on the qualitative results of drug efficacy and disease models, and quantitative indicators based on organ-like gynecological malignant tumor targeting experiments at the level of various types of factors and protein expression levels need more practice and validation. 3) The Frontier Research Cellular Processes Direction lacks an in-depth exploration of molecular pathways that have been studied using organoids, especially common gynecological cancer-related signaling pathways. 4) In this study, only three types of gynecological tumor-like organs were reviewed in detail, i.e., OC-like organs, EC-like organs, and CC-like organs, and few types of tumors were studied.

Future research directions include the following. 1) Investigate the differences in organoid structure or genetic drift in transmission to enhance experimental reproducibility. 2) Highlighting the importance of quantitative results, conducting analysis of actual cell viability, proliferation rate, and other efficacy tests, transforming qualitative research dimensions into quantitative reality dimensions, improving the reliability of organoid application of oncology treatments, and appropriately the incorporation of positive and negative controls, the presence of adequate sample sizes, and the correct statistical test method to increase the accuracy of the assessment. 3) Based on the organ-like model, we will explore the role of common gynecological cancer-related signaling pathways such as PI3K/AKT/mTOR, p53, Wnt, and NF-κB in the progression of gynecological malignant tumors and drug resistance, so as to strengthen the depth of the research direction. 4) Based on OC, EC, and CC, we will carry out specific model construction of different types of other gynecological tumors from the perspective of organ-like, in order to improve the success rate of modelling, study the pathogenesis and therapeutic targets, and achieve the purpose of precision medicine.

## 6 Conclusion

The research described in this review mainly found that OC-like organs were mainly constructed by the matrix gel method, and some studies used the hydrogel method to construct organ-like structures. Both EC and CC organoids were modeled using the matrix gel method. By reviewing three types of gynecological tumors, we found that the organoids of OC, EC, and CC adequately summarize the genetic and morphological characteristics of primary tumors in various subtypes, which can be used for disease modeling, drug detection and screening, patient stratification, and drug response prediction. Modeling gynecological tumor diseases through organoids, replicating the mutation landscape of the primary tumor, and preserving the genomic map of the tumor from which it originated is useful in identifying genes related to OC progression, aiding in disease etiology research, and exploring the mechanisms of gynecological tumor occurrence and development. In addition, constructing different gynecological tumor-like organs can preserving the histological and genetic characteristics of the original tumor as well as tumor heterogeneity, evaluating the efficacy of individual drugs, or conducting drug screening experiments and drug sensitivity tests to explore the mechanisms of drug resistance and drug action in gynecological tumors. Molecular heterogeneity exists in gynecological tumors, leading to differences in drug reactions among different patients, requiring precision medicine. Therefore, organoids that can preserve the heterogeneity and genetic characteristics of the original tumor have advantages in precision medicine. Organ-like models taken from different patient tissues have different drug reactions, and there are broad prospects for stratifying individual patients into customized treatment plans. In summary, the main contribution of this study is the discovery that patient-derived organoids have clinical translational effects in predicting drug responses and resistance mechanisms, and have great potential in precision medicine in the future. Additionally, gynecological tumor organoids provide a choice for *in vitro* personalized simulation of source tissues and organs, which is conducive to promoting the development of clinical personalized precision medicine and regenerative medicine.

On this basis, we point out the dilemmas and difficulties in the existing research and application and propose future directions worthy of research from the perspectives of evaluating the reliability and accuracy of the application of organoids, enriching the types of gynecological tumors studied by organoids, and promoting the translation and application of the research results. It is worth pointing out that although we have recognized the importance of organoids in experimental studies of gynecological malignancies, knowledge of them is still limited, which constrains the development of organelle-based therapeutic agents and treatment modalities, and we need to further explore the optimal modeling methods of gynecological tumor-associated organoids and improve the success rate of modeling, so that it can be used to cultivate more kinds of gynecological tumor-associated organoid models for guiding clinical treatment.

## Figures and Tables

**Table:** 

**CC**	cervical carcinoma
**EC**	Endometrial cancer
**OC**	Ovarian cancer
**3D**	Three- dimensional
**PARP**	poly ADP-ribose
**HRD**	Homologous Recombination Deficiency
**HGSOC**	High-grade serous ovarian cancer
**LGSOC**	Low-grade serous ovarian cancer
**OSE**	ovary surface epithelial
**COL1**	collagen 1
**NRG1**	Neuregulin 1
**SFK**	SRC family kinase
**ECM**	extracellular matrix
**FAK**	Focal Adhesion Kinase
**CDK**	cyclin-dependent kinase
**HRR**	Homologous recombination repair
**MCS**	Multicellular spheroid
**EHS**	Engelbreth-Holm-Swarm
**ECM**	Extracellular matrix
**ERRα**	estrogen-related receptor α
**PDX**	patient-derived xenografts
**Kras**	Kirsten rat sarcoma viral oncogene homolog
**CAF**	Cancer associated fibroblasts
**ASO**	Antisense oligonucleotides
**FGFR**	Fibroblast Growth Factor Receptor
**HPV**	Human papillomavirus
**SCNEC**	Cervical small cell neuroendocrine carcinoma
**SCCC**	Cervical small cell carcinoma
**cCCC**	Cervical clear cell carcinoma
**SqCa**	squamous cell carcinoma
**AdCa**	Adenocarcinoma
**SCJ**	squamocolumnar junction
**CESC**	Cervical squamous cell carcinoma
**LUM**	Lumican
**ACT**	Adoptive T cell therapy
**TIL**	Tumor-infiltrating lymphocytes
**PDTO**	Patient derived tumor organoids
**BME**	Basement membrane extract
**EV**	Extracellular vesicles
**GSC**	Gynecological serous carcinoma
